# Richard Horton. The COVID-19 Catastrophe. What’s Gone Wrong and How to Stop it Happening Again?

**DOI:** 10.1093/eurpub/ckaa216

**Published:** 2020-11-24

**Authors:** Peter Allebeck

**Affiliations:** Department of Global Public Health, Karolinska Institutet, Stockholm, Sweden

Is there anything more one should read about the COVID-19 pandemic? Every day, scientific journals, high-quality magazines and ordinary newspapers are filled with updates, global outlooks and reviews on scientific issues and policy measures related to the pandemic. Yet, this small book of 133 pages gives a perspective we seldom get, in the constant flow of also high-quality information we all are exposed to. As Editor of the *Lancet*, Richard Horton has followed the pandemic from the first reports of cases in Wuhan and publication of the genetic sequence, over clinical trials of vaccines and medicines, to the global debate on control measures and political fights around the WHO. 

‘Why were we not prepared?’ is the title of a chapter that describes how we failed to react on alerts from China and Italy early in the pandemic, how disinformation was spread widely and how many countries looked more to their own interests than global collaboration. He also points out that health services in many countries had suffered from a decade of austerity after the financial crisis.

Horton recognizes impressive scientific efforts and considerable advances made: identification of the virus, genetic sequencing, deployment of testing, hundreds of vaccine trials rapidly in place. But he means that scientific and healthcare leaders should have better understood the magnitude of the problem and given clearer signals to political leaders on appropriate actions. His focus on the British ‘politico-scientific complicity’ is difficult to follow for non-British, but complications in science vs. policy debate, and the difficulty for many politicians to take appropriate actions that are not popular, or illustrate deficiencies in the system, is ubiquitous.

In a chapter titled ‘The politics of COVID-19’, he lists a number of failures in the response of governments to the pandemic: failure in listening to scientific advice, failure to understand and learn from what happened in China, failure in communication to the public, failure in organizing testing and distribution of protective devices. And a fundamental failure of society to address inequalities in society and protect vulnerable people.

Does Horton tell us how to stop it happening again? The final chapter of course does not give any quick fix but urges us to rethink the way we build and organize society. The pandemic does have its origin in a virus, but the dissemination and how it is handled is about behaviour, social life and political structures. Horton reminds us that solidarity, a moral compass and global collaboration are vital for future life and health. The book helps us to take a step back, think about the pandemic in a wider perspective. The true public health view is not only to find out the appropriate control measures, including a new vaccine, but also to engage with political leaders and societal stakeholders, help communicating and help to bridge the science-policy gap.

